# Problem Solving in the Presence of Others: How Rank and Relationship Quality Impact Resource Acquisition in Chimpanzees (*Pan troglodytes*)

**DOI:** 10.1371/journal.pone.0093204

**Published:** 2014-04-02

**Authors:** Katherine A. Cronin, Bridget A. Pieper, Edwin J. C. van Leeuwen, Roger Mundry, Daniel B. M. Haun

**Affiliations:** 1 Comparative Cognitive Anthropology Research Group, Max Planck Institute of Evolutionary Anthropology, Leipzig, Germany; 2 Max Planck Institute for Psycholinguistics, Nijmegen, The Netherlands; 3 Dane County Humane Society, Madison, Wisconsin, United States of America; 4 Max Planck Institute of Evolutionary Anthropology, Leipzig, Germany; 5 University of Portsmouth, Portsmouth, United Kingdom; CNR, Italy

## Abstract

In the wild, chimpanzees (*Pan troglodytes*) are often faced with clumped food resources that they may know how to access but abstain from doing so due to social pressures. To better understand how social settings influence resource acquisition, we tested fifteen semi-wild chimpanzees from two social groups alone and in the presence of others. We investigated how resource acquisition was affected by relative social dominance, whether collaborative problem solving or (active or passive) sharing occurred amongst any of the dyads, and whether these outcomes were related to relationship quality as determined from six months of observational data. Results indicated that chimpanzees obtained fewer rewards when tested in the presence of others compared to when they were tested alone, and this loss tended to be greater when paired with a higher ranked individual. Individuals demonstrated behavioral inhibition; chimpanzees who showed proficient skill when alone often abstained from solving the task when in the presence of others. Finally, individuals with close social relationships spent more time together in the problem solving space, but collaboration and sharing were infrequent and sessions in which collaboration or sharing did occur contained more instances of aggression. Group living provides benefits and imposes costs, and these findings highlight that one cost of group living may be diminishing productive individual behaviors.

## Introduction

Living in a social group confers many advantages, including reduced predation risk and increased learning opportunities [1], [2] yet social life is simultaneously associated with competition for essential resources such as mates and food [3]. Determining which individuals in a social group will obtain limited resources is not straightforward; a suite of individual characteristics probably interacts with a fluctuating social environment to determine access. When alone, individual differences in knowledge and skill may predict resource access, but in a social group there may be additional influential dimensions created by social hierarchies, the knowledge and skill of others, as well as the quality of the social relationships shared between these individuals.

In the wild, chimpanzees (*Pan troglodytes*) are often faced with food resources clumped in space, such as palm oil [4], [5], termites [6], [7], and honey [8], which pose challenging social food-acquisition dilemmas. Chimpanzees live in “fission-fusion” societies in which smaller parties break off from the community and later reunite [6]. Therefore, the exact composition of individuals encountering food resources can vary. Chimpanzees live amidst linear dominance hierarchies which provide some prediction about resource access [6], [9], yet, in some cases, dominance only poorly predicts access to food. Past research on chimpanzees suggests that the nature of the social relationship between individuals [10]–[12], the propensity of low-ranking individuals to innovate to obtain resources [13], and/or differences in skill at obtaining resources [14], [15] can also impact resource acquisition in a social setting.

We presented freely interacting dyads of chimpanzees of known rank and social relationships with a novel resource acquisition task. Chimpanzees were presented with the task both individually and in all possible dyadic combinations within their social group. We first assessed how the presence of a social partner impacted the amount of food obtained compared to when alone, and whether changes in productivity were related to the relative rank of individuals presented with the challenge.

The task used in the current study did not require collaborative solving, nor were the chimpanzees trained to work collaboratively. However, one potential advantage to social living may be the possibility to cooperate with conspecifics to obtain a greater quality or quantity of food over that that could be obtained alone [16], and indeed chimpanzees collaborate in some problem solving experiments in captivity [17], [18] and may forage collaboratively in the wild [19]. Therefore, we were additionally interested in whether a collaborative approach would emerge in some tolerant dyads. Collaboration could take the form of either active coordination or chance occurrence resulting from two individuals being simultaneously attracted to the resource and tolerating the other's presence (“coproduction”) [20]. Finally, we investigated the possibility that individuals could manage to obtain food without solving the task through scrounging or food sharing [19], [21].

## Materials and Methods

### Subjects

The study took place at the Chimfunshi Wildlife Orphanage Trust in Zambia. Fifteen chimpanzees from two social groups (“Group 3” and “Group 4”) took part in this study (see Table 1 for 3-letter ID, age, rank, and sex of subjects from both social groups), forming forty-six unique dyads (one subject, CLE, was not tested in all possible dyads, see below). The social groups were mainly comprised of individuals orphaned as juveniles by hunting or the pet trade, and the groups have been closed to new additions for more than five years. Chimfunshi chimpanzee enclosures are located in miombo woodland forest [22] and the enclosure sizes for Group 3 and Group 4 are 47 and 62 acres, respectively. Chimpanzees remain outside except for 11:30 to 13:30 when they voluntarily enter an attached building for daily supplemental feeding. All sessions took place outside the feeding time when the chimpanzees re-entered the feeding building where the apparatus was located.

**Table 1:** Social group, age, sex and rank of chimpanzees in this study.

Social Group	ID	Age	Sex	Rank in Hierarchy
3	BRI	18	M	2
3	CLE	19	M	3
3	BAR	17	F	5
3	BUF	27	F	5
3	ET	17	F	6
3	LOU	15	F	7
3	BUS	8	M	8
4	VAL	12	M	2
4	NIC	21	M	3
4	SIN	18	M	4
4	BOB	19	M	5
4	MIR	12	F	6
4	KAT	13	F	7
4	BER	12	F	8
4	KIT	7	M	9

### Ethics Statement

Participation by the chimpanzees was voluntary and there were no food or water restrictions applied. This study was non-invasive and strictly adhered to the legal requirements of the country in which it was conducted and the regulations of the Max Planck Institute for Psycholinguistics. This study was approved by the ethical committee of the host sanctuary (the Chimfunshi Research Advisory Board, Project #2011C0040).

### The Tower Task

**Figure 1:**
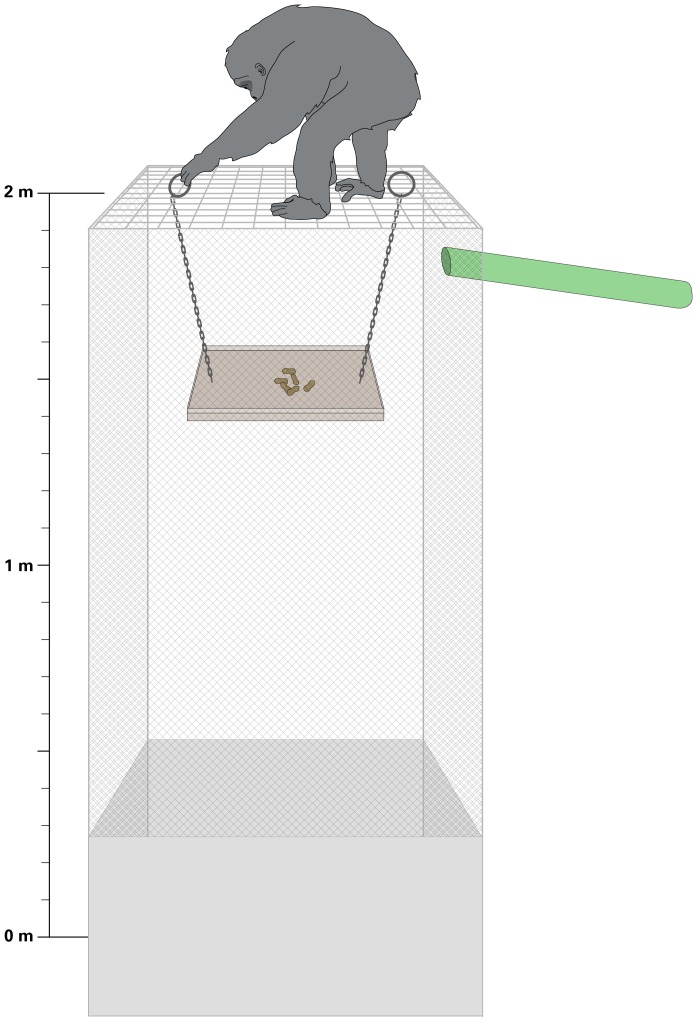
The tower. The tower was constructed of steel mesh and rods that allowed the chimpanzees to see clearly inside the tower. Food was only accessible from the top of the tower if the hanging tray was raised to the top without tipping the tray such that all the food fell to the bottom of the tower. Along the bottom edges of the tower were steel panels to discourage chimpanzees from attempting (in vain) to access fallen food from the ground. The tower was baited through a pipe that extended from the side of the tower to the outside of the room. The size of the chimpanzee is to scale relative to the tower (drawn from photo of adult chimpanzee BOB).

Two identical “towers” were built inside the feeding building, one for each social group. The feeding building was comprised of a series of adjacent rooms, and the towers were located in the first rooms the chimpanzees enter from the outdoor enclosure. The rooms measured 5.4 m×2.5 m×3.5 m (l×w×h), and had barred windows into the enclosure as well as into adjacent rooms inside the building. Chimpanzees entered the room through a sliding door that connected the building to the enclosure, and they could remain in visual, auditory, and limited physical contact with the chimpanzees outside of the building through windows. The towers were made of steel mesh and support bars, and measured 1 m×1 m×2 m (l×w×h). A heavy steel tray (60 cm×30 cm, with a 1 cm high wall around the edge) was suspended inside the tower by two chains; handles were welded on the ends of the chains that extended through the top of the tower. Food was loaded on the tray via a baiting pipe originating outside the room. In order to access the food, chimpanzees had to carefully lift up the two chains such that the tray remained level (otherwise the food would spill into the bottom of the tower where it was inaccessible to the chimpanzees). Chimpanzees had a clear view of whether or not the tray was baited. When the tray was raised to the top of the tower, food could be accessed through openings in a 1 m^2^ area at the top of the tower (Figure 1).

### Learning (Training)

Subjects were initially exposed to the tower individually with the tray suspended just below the top of the tower such that food on the tray was accessible without lifting the tray. Foods were of various types, including pieces of fruits, vegetables, and peanuts. This experience was intended to teach the chimpanzees to expect to access the food from the top of the tower and to increase their comfort climbing on the tower. After they successfully retrieved food from the tray, the tray was lowered gradually over subsequent sessions until the test height of 50 cm was reached (Figure 1). Because it was not possible to fully predict which individuals would enter the testing room, the tray height was typically kept at the easiest height that had not yet been solved by all group members. The handles that extended through the top of the tower were 60 cm apart, enabling a single chimpanzee to reach both simultaneously with some difficulty (requiring the use of hands and feet in order to keep the tray level at the top of the tower while simultaneously obtaining food). Prior to formal testing, chimpanzees had participated in between 6 and 32 (mean 18.5) learning sessions depending on their voluntary participation. Although their skill varied (see Scoring Solo and Dyadic Sessions), only chimpanzees who obtained at least one piece of food from the tray through their own actions (while no other chimpanzee was on the tower) when it was lowered to 50 cm (test height) were included in testing.

### Testing

When a pair or single individual entered the testing room, an experimenter closed the access door connecting the test room to the outside enclosure. When a previously tested individual or dyad entered the testing room, we did not bait the apparatus and left the access door open. A session began when the experimenter baited the tray through an access pipe with six pieces of highly preferred food (hard candies and peanuts). The tray was re-baited (with six pieces) when all food was spilled, when a single piece remained, or when the handles were not touched for at least one minute. If the food was repeatedly spilled or consumed, re-baiting could happen more than once per session. If another consecutive minute passed without any chimpanzee touching the handles, the experimenter did not bait the tray again (i.e., the tray was re-baited only once to try to re-engage). Thus, there was no predefined number of trials or maximum amount of rewards that could be obtained. Sessions were ten minutes long. Chimpanzees were tested once in all dyadic combinations within their group (“dyadic sessions”) and once alone (“solo sessions”). In order to ensure voluntary participation, the order of sessions was not predetermined. All sessions were completed within nine days of testing. Only one subject, CLE, was not tested with all possible partners because he stopped voluntarily entering the building.

### Scoring Solo and Dyadic Sessions

Sessions were videotaped from a custom-made steel camera box fixed inside each test room. From the videos, we coded the exact duration that each chimpanzee spent on top of the tower, both alone and in the presence of their partner (in dyadic sessions), using the software INTERACT (Mangold International GmbH). We also coded the number of rewards obtained by each dyad and individual, and whether the rewards were obtained (a) collaboratively – defined as both individuals simultaneously lifting the handle or chains while food was retrieved from the tray, (b) through scrounging – one individual obtained rewards while the other maintained the height of the tray, or (c) individually – no individual other than the one obtaining food was touching the chains.

We quantified individual skill as the number of rewards each subject obtained during their solo session. Individuals who were quicker to pull the chains and better at keeping the tray balanced obtained a higher score, as they could obtain more rewards in the same amount of time. In Group 3, two females were tested with their dependent young present to avoid stress of separation. The skill, rank and association index of the mother (see Observational Assessment of Social Relations) were used for analyses and rewards obtained by the offspring were attributed to the mother.

To better understand how the presence of a conspecific affected problem solving for food, we coded social behavior during sessions from video. Specifically, we coded all occurrences of contact aggression (push, grab, hit, jump on, bite), non-contact aggression (chasing or lunging, accompanied by fear grimacing or scream), displays (exaggerated movements accompanied by piloerection) and grooming. We coded all instances of begging (reaching with one's hand or mouth for food that is in the possession of another), peering (positioning one's face within a few centimetres of food in the hand or mouth of another), and whether food transfer followed begging or peering. Behavioral definitions were extracted from the most comprehensive ethogram available to date [23], and given that tolerant relationships foster cooperation in some captive settings [18], [24]–[26] we predicted that aggression would be less frequent in sessions in which dyads collaboratively solved the task.

Twenty percent of sessions were randomly selected and independently coded by a second observer for quantity of rewards obtained and method of acquisition. There was a strong correlation between observers for the quantity of rewards obtained per individual (Spearman correlation: r_s_ = 0.994, N = 21). Inter-observer agreement on whether rewards were obtained individually, collaboratively, or through scrounging was also high (Cohen's kappa = 0.916, N = 181). Inter-observer agreement for the occurrence of grooming, requests for assistance, displays, and aggression (combined contact and non-contact) was established by correspondence between coding all sessions from video (KAC) and notes from live observation available from a second observer (BAP) for 31 of 46 dyadic sessions. Agreement for whether or not a session contained these behavioral events was perfect.

### Observational Assessment of Social Relations

Relationship data were extracted from observations of chimpanzees in their social groups outside of the experimental context. The focal follow method at Chimfunshi, initiated in February 2011, consists of daily observations of each group between 8:30 and 11:00 and between 14:30 and 17:00. Focal subjects were selected through systematic, randomized sampling of the chimpanzees' enclosure (as seen from the fence line) and focal chimpanzees were video-recorded for 10 consecutive minutes [27]. Videos were coded in Nijmegen, The Netherlands using the software INTERACT (Mangold International GmbH). For this project we extracted twice-weight association indices [28] generated from one-meter proximity for all dyads from six months of observations. We used 1/0 sampling per dyad per day to assure independence and association indices were generated using the program SOCPROG [29]. Thus, the association measure reflects the frequency with which dyads were found within one meter of each other while in the large outdoor enclosures. Association indices were not available for one individual (LOU) as there was insufficient data due to partial separation from the group for management reasons during earlier months. We obtained measures of rank through independent chimpanzee keeper interviews. The chimpanzee keepers (N = 6) independently agreed on rank order for adult male and female chimpanzees in both groups with the exception of the inversion of one pair of females in Group 3, therefore these females were assigned equal (tied) rank. The keeper ranking was consistent with the direction of submissive signals shown during dyadic sessions. Specifically, all occurrences of potentially submissive gestures were coded from video (including pant grunts, bent wrist offering, crouching, and fear grimacing not preceded by aggression or displays by the other); these behaviors occurred in 22 dyadic sessions and the direction of behavior in these 22 dyads corresponded perfectly with the rank relations obtained from keeper interviews.

### Statistical Analyses

We first assessed whether there was a change in the amount of rewards obtained when another individual was present compared to when individuals were alone, and whether this differed by relative rank, using non-parametric Wilcoxon signed-rank tests. We conducted separate tests for each social group given the widespread evidence of group-level variation in behavior across wild populations [30] and at Chimfunshi [27]. To assess whether social relationships impacted the opportunity for collaboration, we assessed whether the association index was correlated with time spent together on the tower using Mantel tests applied separately to the two groups. The test was conducted with a self-written R script using Spearman's rho as a test statistic and it was exact (enumerating all possible permutations of the data).

We attempted to assess the factors that impacted the rewards obtained by individuals using a Generalized Linear Mixed Model. We assessed model stability by exclusion of the levels of the random effects one-by-one and found the model was unstable with regard to the P-value derived for the full-null model comparison and the estimates derived. We report this attempt in detail in the Supplemental Information (Methods S1) for full transparency, but given the model instability do not pursue this direction further.

To assess whether access to the task was shared, we calculated the percent of sessions in which the task was solved by one individual only (complete monopolization), by both individuals but not at the same time (sequential individual solving), or by neither individual. We calculated the percent of rewards that were obtained collaboratively, through scrounging, and individually. Finally, we report the frequency of aggressive behaviors occurring in sessions that did and did not include collaborative or sequential problem solving. We rely on descriptive reporting given the low frequency of cooperative outcomes in this study. Data will be made readily available to interested parties upon request of the corresponding author.

## Results and Discussion

**Figure 2:**
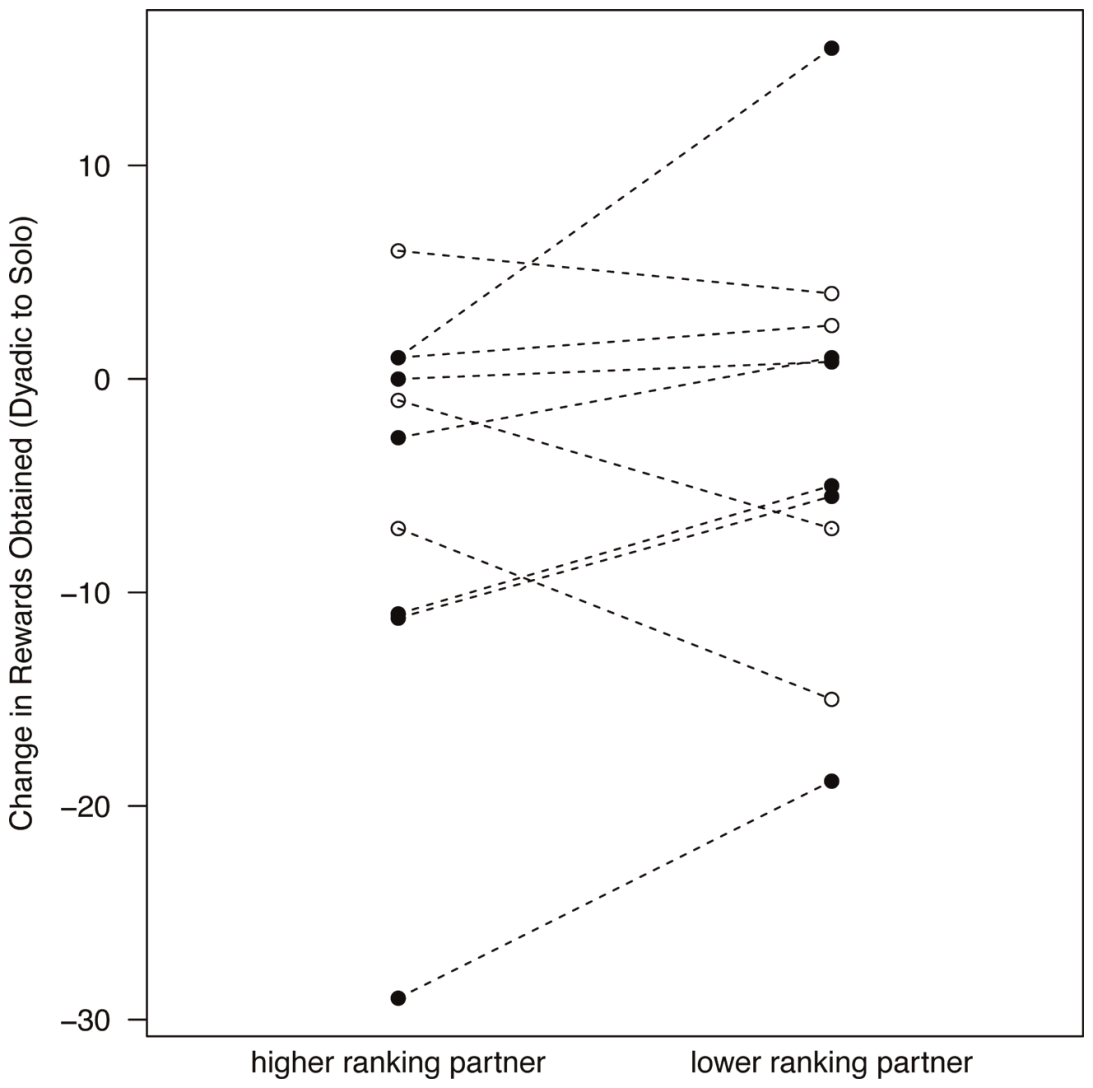
Change in Rewards Obtained from Solo Sessions when in the Presence of Higher and Lower Ranked Individuals. The number of rewards obtained with higher and lower ranking partners was subtracted from the number of rewards obtained in solo sessions. Subjects from Group 3 and 4 are represented by open and closed circles, respectively. Statistics were calculated only on subjects tested with both higher and lower ranking partners in Group 4 (Wilcoxon signed ranks test, W = 21, N = 6, *P* = 0.031).

In solo sessions, chimpanzees spent an average of (X ± SD) 67.4±31.2% of their 10-minute session on the tower and obtained an average of 11.7±9.5 rewards per individual. When tested with another individual in the room, chimpanzees spent 46.6±37.2% of the session on the tower and the average number of rewards obtained per individual across sessions declined to 7.6±8.4 (range 0 to 49; median _solo_ = 12, median _dyadic_ = 6.7, Wilcoxon signed-ranks test, W = 97, N = 15, *P* = 0.035). The magnitude of the loss differed by relative rank in Group 4; compared with solo sessions in which the task was solved by one individual only, subjects lost significantly more rewards when paired with a higher versus a lower ranking partner (Wilcoxon signed-ranks test on subjects tested with higher and lower ranked partners: median change in rewards obtained in dyadic to solo sessions = −7 and −2, respectively, W = 21, N = 6, *P* = 0.031, Figure 2). In Group 3 only four individuals were tested with both higher and lower ranking partners so the loss could not be statistically assessed, however their data are visualized in Figure 2. Therefore, having a social partner present during testing was associated with a decrease in problem solving and reward acquisition. When paired with a higher ranked partner this effect tended to be magnified.

In dyadic sessions, chimpanzees primarily acquired rewards individually. In total, 696 rewards were obtained during 46 dyadic sessions; only seven rewards were obtained collaboratively (which occurred in three dyads for a total of 1% of rewards), 13 by scrounging (which occurred in seven dyads for a total of 1.9% of rewards), and the remaining 97.1% of rewards were obtained individually. Thus, collaboration was extremely rare. These findings are consistent with a growing body of evidence indicating that when chimpanzees do not absolutely require a partner in order to obtain resources in experimental tasks, they tend to work alone [31], [32].

**Figure 3:**
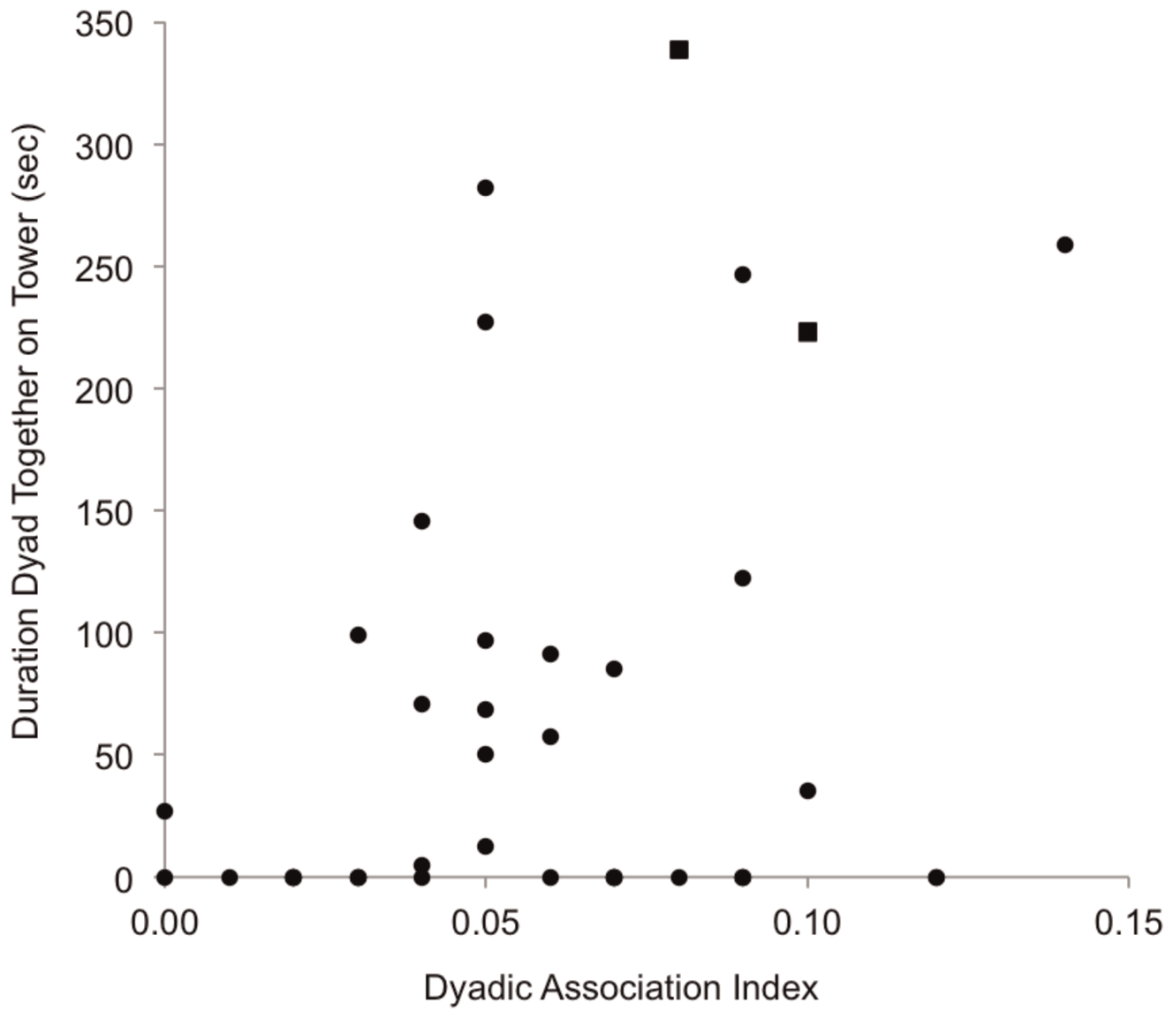
Dyadic Association Index & Time Shared on Tower. The y-axis shows the duration in seconds in which both chimpanzees were located on top of the tower during dyadic sessions. The x-axis shows the twice-weight association index generated from six months of focal follows. Dyads that showed at least one occurrence of cooperative problem solving are represented by a square (with the exception of the dyad LOU & BUF because no AI was available). In both social groups, there was a significant positive correlation between AI and time shared on the tower (Mantel tests, Group 3: r_S_ = 1, N = 6, *P*<0.01; Group 4: r_S_ = 1, N = 8, *P*<0.01).

Collaboration, whether coordinated or an outcome of individual actions, required two individuals to be simultaneously situated on top of the tower. In dyadic sessions, two individuals simultaneously occupied the tower for an average of 11.9±17.2% of sessions (N = 46; range 0 to 58.3%), or 1.2 minutes of a ten-minute session. Thus, opportunities for collaboration were infrequent given that dyads rarely occupied the tower simultaneously. All three dyads that demonstrated collaborative solving spent more time together on top of the tower than the average dyadic shared time in this experiment (LOU & BUF: 58.3%, VAL & KAT: 56.4%, KIT & KAT: 37.2%). Finally, in both groups, there was a significant correlation between dyadic association index and time shared on the tower (Mantel tests, Group 3: r_S_ = 1, N = 6, *P*<0.01; Group 4: r_S_ = 1, N = 8, *P*<0.01, Figure 3).

To assess whether access to the task was “shared” (that is, whether more than one individual solved the task in a dyadic session), we categorized dyadic sessions based on how many chimpanzees solved the task individually within a session. One individual solved all trials (complete monopolization) in 37 sessions (80.4%), both individuals solved the task (sequentially) in five sessions (10.9%), and neither individual solved the task in four sessions (8.7%). The five dyads that engaged in sequential individual solving were comprised of nine individuals (4 females, 5 males) and three of the five dyads included the youngest individual tested in each group. Of the three dyads reported above to show collaborative problem solving, two also showed sequential solving in the same session (VAL & KAT, LOU & BUF).

Contact aggression occurred in seven dyadic sessions (15.2%) by nine different individuals; in two cases it was shown by both individuals in the dyad and in the remaining five it was unidirectional. In all unidirectional cases, contact aggression was directed from higher toward lower ranking individuals and the aggressor obtained more rewards in that session. Noncontact aggression occurred in five sessions, only one of which did not contain contact aggression. Thus, considering contact and non-contact aggression together, 8 of 46 sessions contained aggression (17.4%). Displays were more frequent, occurring in 16 sessions (34.8%), demonstrated by 13 of the 15 subjects. Social grooming occurred in two dyadic sessions (4.3%) by three individuals, and in no case did the groomer obtain any rewards in that session. Thus, there was no evidence of grooming to obtain access.

Table 2 indicates the distribution of aggressive events (contact and noncontact) across sessions that did or did not involve collaborative problem solving (Table 2) and shared access to the tower (Table 3). Aggression occurred in 67% of the sessions with collaborative problem solving and 13% of sessions without. Aggression occurred in 40% of the sessions that contained sequential solving and 15% of sessions without. Therefore, aggression appeared to be more frequent when both attempted to engage in the task, either simultaneously or sequentially. Although descriptive, these data are in the opposite direction of what we predicted. Here we see that cooperative outcomes (encompassing any scenarios in which two individuals solved the task within the same session) were associated with more aggressive events. This suggests that the chimpanzees were not strategically converging on a cooperative solution, but rather were individually competing for access.

**Table 2:** Frequency of sessions with and without aggression, categorized by whether collaborative problem solving occurred in the session.

	Aggression	No aggression	Total
Collaborative problem solving occurred	2	1	3
Collaborative problem solving did not occur	6	37	43
Total	8	38	46

**Table 3:** Frequency of sessions with and without aggression, categorized by whether one chimpanzee, two chimpanzees, or neither chimpanzee solved the task in the session.

	Aggression	No aggression	Total
One individual solved	6	31	37
Two individuals solved sequentially	2	3	5
Nobody solved	0	4	4
Total	8	38	46

In the majority of sessions only one individual obtained benefits and the other individual, who had the knowledge and skill to solve the task, inhibited his or her own motivation to solve it and effectively avoided aggressive encounters. The behavioral results suggest that inhibition could be an important response to learn in order to maximize one's fitness in natural settings, as the cost incurred by aggression was probably evaluated to be greater than the benefits received by solving the task for some individuals in some social pairings. Therefore, temporary inhibition may be a beneficial strategy for a chimpanzees given that they live in fission-fusion societies with temporary party formations and resources that vary in spatial and temporal distributions [33].

Of the 13 rewards that were scrounged, five were obtained by KIT, a seven-year old male who was the youngest independent subject, and six were obtained by dependent young (scrounged from non-mother). The remaining two scrounging events were by adults. In contrast to scrounging in which one chimpanzee reached for food on the tray, we also coded for begging directly from another individual already in possession of the food. Begging was rare and never successful. Only one individual begged (the juvenile KIT) and in no case was begging followed by food transfer. Peering, a behavior in which individuals move their face very close to the food in possession of another but do not reach for it, was shown by four individuals (two juveniles and two adults) but never resulted in food transfer.

Thus, scrounging and begging were infrequent and not productive strategies, accounting for less than 4% of rewards obtained. The low frequency of scrounging and begging may have been due to the limited space on top of the tower; the positive relationship between the association index and time shared on the tower suggests that this limited space was below the comfort threshold for many dyads, at least when food was present [34], [35]. Scrounging or begging for food may be a risky behavior depending on the reaction of the food possessor, and the risk may have been greater in the limited space on the tower.

One limitation of this study is that social sessions were conducted in dyads while parties of three or more were not assessed. While this allowed us to systematically assess how dyadic relationship measures impact problem solving, this approach does not explore how the presence of coalition partners [36] or those with whom a close social bond is shared [37], may have influenced behaviour in the presence of a third party. Given the complexity of chimpanzee social relations, this would be an interesting future direction to pursue as it may reveal more flexibility in the way chimpanzees can approach social challenges over resources.

It is possible that some dyads would have been more likely to approach this problem collaboratively or share access to the tower if they did not have previous experience solving the task alone. Given that their initial success at the tower during learning sessions did not involve the presence of another, perhaps they perceived the task as an individual challenge and when another chimpanzee was present they viewed the other as an impediment to their individually-learned solution. Determining how previous individual and collaborative experience impacts the perception of new challenges (as either collaborative or individualistic) would be interesting to explore as another factor that may predict the emergence of collaboration and sharing in primates.

## Conclusions

Chimpanzees fared less well in the presence of others compared to when they were able to work independently. This was not due to two individuals accessing the spoils and, in consequence, receiving less as an individual, but to an on-going struggle amongst the two individuals negotiating access to the tower. The ability of chimpanzees to inhibit their behavior is striking in this study; individuals with the necessary skill to obtain rewards often abstained from approaching the tower and solving the task when others were present (see also [38]). Given that aggression occurred in sessions where both individuals attempted to access rewards, inhibition may be the best strategy for an individual who may be in the company of lower ranking individuals at a later time, or have access to an alternative food source. Chimpanzees tended to solve this novel resource acquisition task alone; one individual in each dyad monopolized the majority of the resource and individuals largely avoided simultaneously occupying the space required to solve the problem. These findings demonstrate that chimpanzees probably consider both the potential energetic gains of accessing a resource and the potential physical costs of aggression by conspecifics when deciding whether or not to solve a foraging problem in the presence of others, and highlight the complex social landscape that is navigated by group-living species when making foraging decisions.

## Supporting Information

Methods S1 **Details of generalized linear mixed model instability.**
(DOCX)

Methods S2 **ARRIVE Checklist.** Document indicating how the present study adheres to the ARRIVE (The Animal Research: Reporting of In Vivo Experiments) guidelines, based on the initiative of the UK National Centre for the Replacement, Refinement and Reduction of Animals in Research. The goal of the guidelines is to improve consistency in reporting.
(DOC)
