# Supplementary material for: Problem Solving in the Presence of Others: How Rank and Relationship Quality Impact Resource Acquisition in Chimpanzees (Pan troglodytes)
Source: PLoS One. 2014 Apr 2;9(4):e93204. doi: 10.1371/journal.pone.0093204 (PMC3973697; doi:10.1371/journal.pone.0093204)
Supplement: Methods S1 — Details of generalized linear mixed model instability. (DOCX) [file pone.0093204.s001.docx]

Supporting Information

Methods S1

We attempted to assess the factors that impacted the rewards obtained by individuals using a Generalized Linear Mixed Model [GLMM; 1] that included rank distance, group membership, the skill of the subject and partner, the two-way interactions between rank distance, and the two skill measures as fixed effects. We additionally included dyad, subject and partner as random effects. We included random slopes components [2] and the total sample size was 92 data points (46 dyads formed out of 15 subjects). As an initial overall test we compared the full model with a null model comprising only subject skill and all random intercept and random slope effects [3] using a using a likelihood ratio test [4]. We assessed model stability by exclusion of the levels of the random effects one-by-one and found the model was unstable with regard to the P-value derived for the full-null model comparison and also the estimates derived (range in P-values 0.029 to 0.571). After removal of the non-significant interactions, the model remained unstable. There was no obvious effect of the predictors as a whole on the response and neither of the two interactions nor the effect of group revealed significance but this result was associated with considerable uncertainty. We report this attempt in an effort to provide full transparency, but given the model instability do not pursue this direction further.

1. Baayen RH (2008) Analyzing Linguistic Data: Cambridge University Press.

2. Barr DJ, Levy R, Scheepers C, Tily HJ (2013) Random effects structure for confirmatory hypothesis testing: Keep it maximal. Journal of Memory and Language 68: 255-278.

3. Forstmeier W, Schielzeth H (2011) Cryptic multiple hypotheses testing in linear models: overestimated effect sizes and the winner's curse. Behavioral Ecology and Sociobiology 65: 47-55.

4. Dobson AJ (2002) An Introduction to Generalized Linear Models. CRC, Boca Raton: Chapman & Hall.
